# Antenatal Classes in the Context of Prenatal Anxiety and Depression during the COVID-19 Pandemic

**DOI:** 10.3390/ijerph19095073

**Published:** 2022-04-21

**Authors:** Aleksandra Ciochoń, Anna Apanasewicz, Dariusz P. Danel, Andrzej Galbarczyk, Magdalena Klimek, Anna Ziomkiewicz, Urszula M. Marcinkowska

**Affiliations:** 1 Department of Environmental Health, Institute of Public Health, Faculty of Health Sciences, Jagiellonian University Medical College, 31-066 Kraków, Poland; aleksandra.ciochon@doctoral.uj.edu.pl (A.C.); a.galbarczyk@uj.edu.pl (A.G.); magda.klimek@uj.edu.pl (M.K.); ummarcinkowska@gmail.com (U.M.M.); 2 Department of Anthropology, Ludwik Hirszfeld Institute of Immunology and Experimental Therapy, Polish Academy of Sciences, 53-114 Wrocław, Poland; anna.apanasewicz@hirszfeld.pl (A.A.); dariusz.danel@gmail.com (D.P.D.); 3 Laboratory of Anthropology, Institute of Zoology and Biomedical Research, Jagiellonian University, 30-387 Kraków, Poland

**Keywords:** breastfeeding, infant development, maternal mental health, pregnancy during the COVID-19 pandemic

## Abstract

Perinatal maternal anxiety and depression negatively affect intrauterine fetal development, birth outcome, breastfeeding initiation, duration, and milk composition. Antenatal classes potentially reduce the anxiety of pregnant women and may thus contribute to healthy infant development. The study investigates the relationship between participation in online or in-person antenatal classes and levels of anxiety and depression in Polish women during the COVID-19 pandemic. The study group included 1774 adult, non-smoking pregnant women. We compared the state anxiety (STAI-State) and depression levels (EPDS) in women who (i) attended antenatal classes in-person, (ii) attended online classes, and (iii) did not attend any of them. The statistical analyses included a GLM model and trend analysis, while controlling for maternal trait anxiety, age, pregnancy complications, trimester of pregnancy, previous pregnancies, and COVID-19 infections. We observed statistically significant differences in the level of anxiety (and depression). Women who did attend antenatal classes in person had the lowest levels of anxiety and depression. Considering the importance of maternal mental well-being on fetal development, birth outcome, and breastfeeding, in-person participation in antenatal classes should be recommended to pregnant women.

## 1. Introduction

Pregnancy and the perinatal period including breastfeeding is a challenging time for many women who experience numerous physiological and sociological changes. The most common reasons for worries include in utero fetal health, possible delivery complications, and potential problems associated with maternal care [1]. Factors such as maternal age, pregnancy planning, traumatic experiences during previous pregnancy or delivery, medical complications during current pregnancy, as well as previous mental disorders not associated with pregnancy including anxiety and depression might predispose women to high prenatal anxiety [2,3].

Increased maternal anxiety, as well as prenatal depression induce negative consequences for pregnancy outcomes including risk of miscarriage, preterm delivery, C-section, and low infant birth weight [3,4,5]. Studies show that maternal anxiety during the perinatal period also affected breastfeeding. High maternal anxiety is related to lower breastfeeding intention and exclusivity [6], decreased rate of breastfeeding [7], and shorter duration and earlier termination of breastfeeding [8]. Moreover, maternal distress and anxiety is also associated with breast milk composition, negatively affecting its nutritional and immunoactive properties. High maternal stress decreases milk energy density and fat content and changes fatty acid profile [9,10]. It also decreases levels of milk secretory immunoglobulin A and lactoferrin [10,11,12].

Antenatal classes, which aim to transfer practical knowledge about labor, puerperium, breastfeeding, and early parental care, have the potential to decrease maternal anxiety and thus reduce potential risks for breastfeeding and infant development [13,14,15,16]. Studies on antenatal education have shown that participation in classes improves the general mood and well-being of pregnant women, therefore, they can endure labor pain more effectively [15]. Moreover, women attending antenatal classes during pregnancy cope better with pregnancy-related stress during the perinatal period compared to those who received only routine medical care [17]. Participating in antenatal education also decreases risk of breastfeeding cessation during the first month after parturition [16,18] and increases maternal confidence [19] and breastfeeding self-efficacy [20,21].

In addition to an educational role, antenatal classes also fulfill a crucial psychosocial role. The possibility of exchanging information and experiences between parents, under the guidance of a specialist, provides support to the future mother and helps her cope with the upcoming changes. It is worth mentioning the role of the child’s father, who can also participate in these activities. The perspective of sharing a mother’s dilemmas with a partner in a supportive environment allows her to experience the time of pregnancy, childbirth, and breastfeeding in an atmosphere of mutual respect and understanding [13,14], and contributes to her better physical and mental well-being. In accordance, support from partners significantly influences women’s decision to initiate and continue breastfeeding [22,23], and father’s participation in antenatal education is associated with an improved rate of any breastfeeding at six weeks after parturition [24]. 

Health policies for pregnant women differ between countries. Despite many advantages, participation in antenatal classes is not obligatory in Poland. Due to the limited opportunities (a small number of free public institutions offering classes), pregnant women frequently decide to participate in paid classes, which involves additional costs. Unfortunately, not every pregnant woman can afford such an expense [14,15,16,17]. 

The COVID-19 pandemic is currently one of the most serious public health problems worldwide with various psycho-emotional disturbances for pregnant women [25]. Pregnancy, childbirth, and breastfeeding during the pandemic represent an additional burden and challenge for women, associated with intensified fear about the health and life of the baby, the course of labor and the entire hospitalization [26], and safety of breastfeeding in the face of possible infection. A study in Ireland found that, since the outbreak of the pandemic, more than 50% of pregnant women always worry about their health, and as many as 35% have changed their daily habits. Due to the fear of potential infection and introduced restrictions, women avoided social contact, did not use public transport, and were more willing to work and do their shopping remotely [27].

Recently, some parts of antenatal and perinatal maternity care have moved to the internet or were done by phone. Previous research found that care in the form of phone calls reduces pregnant women’s feelings of anxiety by an average 5.8 points compared to women who did not receive this form of education and counseling (tele-education: 24.25 ± 4.90, no tele-education: 30.04 ± 8.48; *p* < 0.001) [28]. However, there is a lack of knowledge if online antenatal classes are as helpful for parents as in-person classes. This study aimed to compare levels of anxiety and depression during pregnancy between (a) participants of online antenatal classes, (b) participants of in-person classes, and (c) pregnant women who did not participate in any kind of antenatal education during the COVID-19 pandemic.

## 2. Materials and Methods

### 2.1. Project Description

The presented study is a part of the ongoing, longitudinal *Corona Mums Project* conducted since May 2020 among women during the pregnancy and postpartum period. The project’s recruitment and information were advertised via Polish social media groups (mainly via Facebook) for pregnant women including *Mleko Mamy +, Położna radzi,* and *Rodzimy razem* (all in Polish). We also advertised the study on the project fan page: https://www.facebook.com/mamawkoronieUJ/ (accessed on 19 June 2020), on the radio broadcasts (*Radio Rodzina, ESKA,* and *Radio Kraków*), and in the newspapers (*Gazeta Wyborcza* and *Dziennik Polski*) and magazines about motherhood (*Mamo to ja*). The study included an online questionnaire, collecting information about demographics (place of residence, socio-economic status, etc.), mental health assessments, course of pregnancy (including possibility and form of participation in antenatal classes), infection with COVID-19 (self-reported), and complications of pregnancy (such as anemia, diabetes, and preeclampsia). A large part of this questionnaire, except questions about COVID-19 infection and antenatal education, was used in a previous study conducted in lactating women [10,29]. The research was anonymous, and to avoid duplicate responses filling in questionnaires twice from a device with the same ID was blocked.

The study protocol was approved by the Bioethics Committee of the Jagiellonian University (date: 16.12.2020 r., decision number: 1072.6120.359.2020). Informed, written consent was obtained from all participants of the study. 

### 2.2. Study Group

The study group included 1774 women from Poland, aged 19–43 (mean age = 30.26 years; SD = 3.76), non-smokers, who were in the second or third trimester of pregnancy during the COVID-19 pandemic. All participants filled in the online questionnaire (complete information regarding all measured values) between May 2020 and March 2021. Due to the relatively small number of pregnant women in the first trimester (N = 242) and their low interest in participating in antenatal classes (the majority of them had not decided whether to participate), we decided to exclude women in the early stage of pregnancy from the analysis.

### 2.3. Participation in Antenatal Classes, Anxiety, and Depression

Pregnant women were asked if they participated in antenatal classes and could choose one of three options: (a) yes, I did participate in the classes in-person; (b) yes, I did participate in the online classes; or (c) no, I did not participate in any form of the classes.

State anxiety (STAI-State) and trait anxiety (STAI-Trait) was assessed based on the Polish version of the State-Trait Anxiety Inventory (STAI) [30]. This questionnaire is based on a 4-point Likert scale and includes 40 questions [31]. 

The Polish version of the Edinburgh Postnatal Depression Scale (EPDS) was used to estimate the prevalence of depressive symptoms during pregnancy [32]. Even though this questionnaire is intended for the postpartum period, it is widely used as a tool to assess depression antenatally. Moreover, due to the longitudinal character of the study with a prepartum and postpartum assessment, it was chosen as the most comprehensive one for repeated measurements. This questionnaire includes 10 items assessing the feelings of self-blame, anxiety, panic attacks, and suicidal thoughts during the previous week [32]. The Polish translation for the both questionnaires is characterized by high validity and reliability (The Cronbach’s alphas: STAI-State: 0.90; STAI-Trait: 0.88; and EPDS: 0.91) [30,32]. The scores based on questionnaires received in our study were not used for diagnostic purposes.

### 2.4. Statistical Analysis

We used general linear modeling (GLM model) to investigate if different forms of participation in antenatal classes (3 categories) are related to anxiety (STAI-State) and depression (EPDS) while controlling for maternal trait anxiety, age, pregnancy complications (yes/no), trimester of pregnancy (second trimester/third trimester), previous pregnancies (primipara/multipara), and COVID-19 infections (yes/no). Post hoc pairwise comparisons were conducted using Bonferroni correction. Additionally, we employed planned contrast analysis and tested linear trends in the intensity of the depression and anxiety symptoms in women from different study subgroups. A probability value of *p* < 0.05 indicated statistically significant results. The analyses were performed in SPSS software, version 27 (Chicago, IL, USA).

## 3. Results

Out of 1774 pregnant women participating in the study, 858 (48.5%) were primiparous and 916 were multiparous. Pregnancy complications occurred in 421 (23.7%) women, 122 were diagnosed with COVID-19 (6.9%), and 673 (37.9%) women were in the second trimester. Among all surveyed women, 633 women (35.7%) participated in antenatal classes in-person, 427 women (24.1%) in online classes, and 714 women (40.2%) did not participate in any classes. Descriptive statistics for each group can be found in Table 1. 

Both the models for anxiety (F_15,1771_ = 118.98; *p* < 0.001) and depression (F_14,1771_ = 3.58; *p* < 0.001) were statistically significant. After controlling for the confounding factors (maternal age, pregnancy complications, STAI-Trait, COVID-19 infection) the analysis showed significant differences in the levels of state anxiety and depression. The associations between studied factors and the STAI-State and EPDS values are presented in Table 2. The lowest STAI-State and EPDS scores were noted in women who attended in-person classes (STAI: mean = 42.20, SD = 10.61; EPDS: mean = 7.41, SD = 4.96), the intermediate in participants of online classes (STAI: mean = 44.62, SD = 10.24; EPDS: mean = 8.28, SD = 4.95), and the highest in those who did not attend birthing school at all (STAI: mean = 46.18, SD = 11.23; EPDS: mean = 8.96, SD = 5.35).

**Figure 1:**
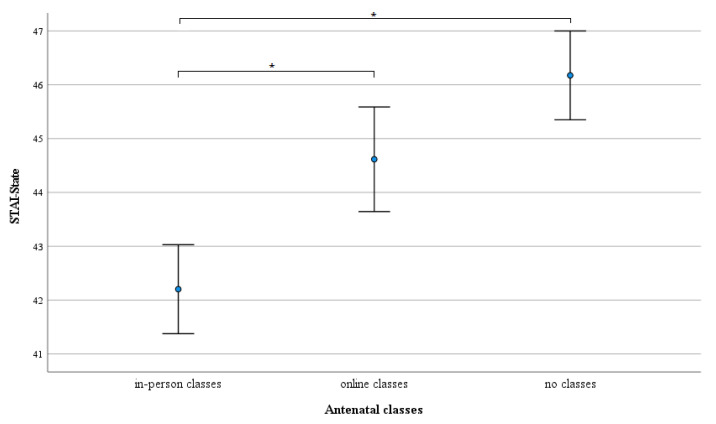
Differences in mean levels of state anxiety (dots) between in-person antenatal classes participants, online antenatal classes participants, and non-participants of any kind of antenatal classes adjusted for maternal age, pregnancy complications, COVID-19 infection, and STAI-Trait. Whiskers show 95% CI, * *p* < 0.05.

Post hoc pairwise comparisons with the Bonferroni correction showed statistically significant differences in STAI-State between women attending in-person classes and those who attended the online classes (*p* = 0.002), as well as women who did not attend antenatal classes (*p* < 0.001). The difference in STAI-State reported by women who took online classes and those not attending antenatal classes were not statistically significant (*p* = 0.429) (Figure 1).

**Figure 2:**
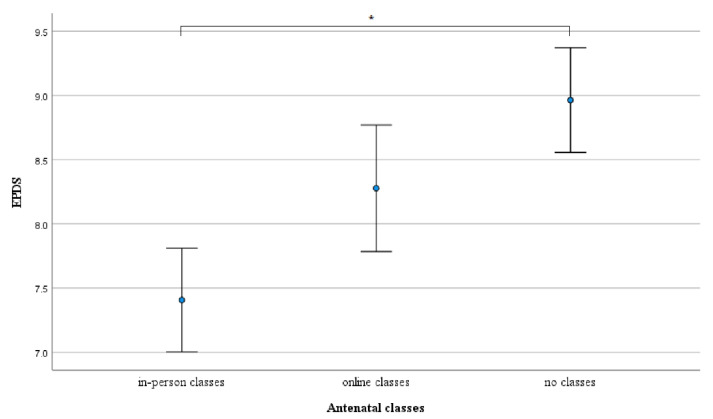
Differences in mean levels of depression (dots) between in-person antenatal classes participants, online antenatal classes participants, and non-participants of any kind of antenatal classes adjusted for maternal age, pregnancy complications, and COVID infections. Whiskers show 95% CI, * *p* < 0.05.

The difference in EPDS Scores between women who attended in-person classes and those women who did not take antenatal classes was statistically significant (*p* < 0.001). Nonetheless, no statistically significant differences were found between women taking in-person classes and online classes (*p* = 0.085), and between women attending online classes and women who did not participate in antenatal courses (*p* = 0.214) (Figure 2).

The observed trends in the increase in both STAI-State (F_1;1771_ = 17.089; *p* < 0.001) and EPDS Score (F_1;1771_ = 6.963; *p* < 0.01) associated with non-participating vs. participating different forms of classes were statistically significant, demonstrating the significant differences between the highest scores among non-participants and the lowest scores among in-person participants of antenatal classes. Referring to other variables, we did not observe a significant effect of COVID-19 infection, parity, maternal age, and pregnancy trimester on STAI-State. Pregnancy complications and STAI-Trait were associated with increased STAI-State (Table 2). The whole model, including antenatal classes participation, accounted for nearly 51% of the variability in STAI-State.

Similarly, COVID-19 infection and parity had no effect on EPDS score. In contrast, pregnancy complications, maternal age, and the pregnancy trimester were associated with the EPDS score. The whole model, including antenatal classes participation, accounted for 3% of the variability in EPDS score.

## 4. Discussion

The analysis revealed that pregnant women who attended in-person antenatal classes had significantly lower levels of anxiety compared to women who attended online antenatal classes or did not attend these classes. In addition, pregnant women who attended in-person classes had a lower score of EPDS compared to those not participating in classes. The increased trait of anxiety and presence of pregnancy complications were both associated with increased levels of prenatal anxiety, while maternal age, presence of pregnancy complications, and trimester of pregnancy heightened the risk of prenatal depression. Neither COVID-19 infection nor parity had any effect on anxiety levels and risk of depression. 

The spread of the SARS-CoV-2 virus and the increased mortality associated with contracting COVID-19 led to the implementation of severe restrictions involving isolation and social distancing in many countries around the world. Pregnant women usually face increased anxiety and risk of depression due to physiological changes, worries about the child’s prenatal development and health, course of delivery, and the postpartum period. During the pandemics, women have been exposed to additional worries associated with limited access to medical care and the potential impact of COVID-19 infection on the unborn child. After delivery, infected women are frequently separated from their newborns due to hospital regulations, which further increases maternal anxiety, hinders mother–infant bonding, and decreases the chance for successful breastfeeding initiation. 

Many pregnant women facing worries and problems during pregnancy look for information about childbearing, healthcare, and support from specialists and other mothers while participating in antenatal classes [27,33]. Previous studies showed that participation in antenatal classes is beneficial for pregnant women’s well-being by reducing anxiety and the risk of postnatal depression [15,16,34], especially when these classes included respiratory exercises, mindfulness practices, and learning to cope with anxiety [15,35]. However, due to the pandemic restrictions that were introduced in many countries, including Poland, since March 2020, pregnant women could not or decided not to attend antenatal classes in-person. Instead, nearly one in four gravidas participating in our project decided to attend online classes that were perceived as easily accessible and safe by means of not increasing the risk of infection. Online classes were also recommended in other countries and became an increasingly popular form of antenatal care [36]. 

A previous study suggested that online antenatal classes can also reduce prenatal anxiety, however, the authors did not compare those results to in-person classes, but solely to no classes at all [37,38,39]. Thus the effectiveness of online classes, when compared to in-person participation, remains questionable. Taking into consideration the increasing popularity of online antenatal classes and the importance of maternal psychological well-being for a child’s prenatal and postnatal development and breastfeeding, determination of the effectiveness of online classes seems to be essential [40]. Our research answers this need by demonstrating that in-person participation in antenatal classes is superior to online classes when it comes to reducing maternal anxiety level. 

Prenatal depression results in a number of health issues for children and mothers. For children, it increases the risk of premature birth and fetal growth restrictions [41]. For mothers, it is associated with complications during pregnancy and parturition, “postpartum blues”, and postpartum depression [34,42,43,44]. Prenatal and postnatal depression is also associated with a lower rate of breastfeeding [45], and exclusive breastfeeding duration [40]. Previous studies have shown that the risk for prenatal depression during the COVID-19 pandemic is doubled compared to pre-pandemic levels [46]. In our study, we observed a reduced EPDS score among pregnant women who attended antenatal classes in-person, compared to non-attenders. We also found that the intensity of depression symptoms in online participants did not differ significantly from the non-participants. This observation stands in contrast to the results of a quasi-experimental study from Taiwan, where e-health, web-based antenatal education was associated with lower pregnancy stress and higher self-efficacy than standard education delivered to pregnant women in-person [47,48]. However, it has to be acknowledged that, in the case of this study, routine education was delivered individually. Such a solution excludes the positive effect of peer support from other pregnant women, who co-participate in antenatal classes in Poland. Lack of peer social support may thus explain differences in results between Tsai et al. [49] and our study.

The observed differences in the level of anxiety and depression symptoms between non-participants and participants of different forms of education might have several explanations. First, it is possible that more anxious women were stricter in social distancing, and they preferred staying at home, not participating in antenatal education or choosing online classes. Indeed, studies demonstrate that people with high anxiety and COVID-19 infection fear were predisposed toward more self-care and avoidance behavior [27,42,44,45,46,47]. The strong effect of trait anxiety found in our study suggests that this explanation is plausible. A study with repeated surveying of each participant could aid in testing this hypothesis. 

It is also probable that online classes were not as effective as the on-site classes in teaching relaxation. As a consequence, online participants could have encountered problems utilizing relaxation methods in practice, especially as even participants of traditional classes sometimes report problems implementing these methods [15]. 

Finally, antenatal classes also play a psychological function of peer support. During in-person antenatal classes, women can meet other future mothers and bond with them. Finding support among other antenatal class participants improves well-being and is beneficial to women’s mental health after delivery [48]. Social support was found to be one of the principal factors in decreasing maternal anxiety and depression during the postnatal period. It is also associated with increased birth weight [37,41], and higher levels of immunoactive factors in breast milk [29]. Social distancing and online participation in antenatal classes do not facilitate making new bonds. Although there is a general lack of knowledge about establishing emotional connections among online antenatal classes attendees, university students after online classes reported a sense of loneliness, low social support, and problems with maintaining social networks [47,50,51]. 

It is unfortunate that during the sanitary restrictions, when most activities are transferred to the virtual world, there are some indications that online classes do not improve the mental well-being of pregnant women as much as in-person classes would. Low maternal psychological well-being during pregnancy may result in higher levels of anxiety and increased depressive symptoms. Thus, it can be associated with less favorable developmental conditions during the prenatal period [3,4,5]. Postnatally, it may result in lower maternal–infant bonding and a lower chance for breastfeeding initiations and a shorter breastfeeding duration. In that perspective, attending in-person antenatal classes associated with lower maternal anxiety and depression symptoms whenever possible would be more beneficial for a child’s development than attending the online substitute. 

Our results should be interpreted in light of some limitations. Firstly, although results of our analyses were statistically significant, the overall observed effect size was not large, which can indicate a relatively low difference in anxiety and depression symptoms between participants of various forms of antenatal education. Secondly, we did not collect data on the quality of the classes the participants attended, either online or in person. Hence, in future studies, attendees’ opinions and the received social support should be included in the questionnaire to adequately assess the quality of online antenatal classes. Another limitation is the risk of self-selection and external selection resulting from the level of education of participants or access to online resources. Unfortunately, we do not have information on how many pregnant women participating in the study had symptoms of depression or anxiety before pregnancy. The lack of this information is the next limitation of our study. A final limitation worth mentioning is including women who delivered only a complete set of data. In our study, women who did not complete a significant portion of the questionnaire were excluded from the analysis. Thus, the 1774 participants whose results were statistically analyzed have complete information on all measured values. The use of the complete cases approach may also introduce errors depending on the mechanism of missing data. 

## 5. Conclusions

Maternal prenatal anxiety and depression have a significant impact on breastfeeding and child development during prenatal and postnatal periods. The opportunity to attend antenatal classes is vital in terms of reducing anxiety and depression symptoms and maintaining a positive sense of well-being in pregnant women.

In our research, we observed increased levels of state anxiety in women who did not attend antenatal classes compared to those attending online and in-person classes. Women who did not attend antenatal classes had the highest levels of anxiety and depression. To reduce maternal anxiety and the risk of depression, participating in online and in-person antenatal classes is preferable to not attending.

Due to the better mental well-being of pregnant women attending in-person classes compared to online classes, it should be recommended to use this form of antenatal classes whenever health risks do not outweigh the benefits associated with it.

It is also worth considering the preparation of appropriate psychological support programs that could be presented in social media and traditional media, increasing social awareness of the studied issue.

## Figures and Tables

**Table 1:** Descriptive statistics of the variables in the study groups.

Variables	Attending Antenatal Classes	Mean	SD
Age (years)	In-person classes	30.22	3.66
	Online classes	29.71	3.53
	No classes	30.61	3.94
STAI-State	In-person classes	42.2	10.61
	Online classes	44.62	10.24
	No classes	46.18	11.23
STAI-Trait	In-person classes	43.69	8.47
	Online classes	44.76	8.21
	No classes	46.17	8.49
EPDS	In-person classes	7.41	4.96
	Online classes	8.28	4.95
	No classes	8.96	5.35
		N	%
Pregnancy complications			
Yes	In-person classes	247	13.9
	Online classes	106	6
	No classes	68	3.8
No	In-person classes	212	12
	Online classes	427	24.1
	No classes	714	40.2
COVID-19 infection			
Yes	In-person classes	52	2.9
	Online classes	23	1.3
	No classes	47	2.7
No	In-person classes	581	32.7
	Online classes	404	22.8
	No classes	667	37.6
Trimester of pregnancy			
Second	In-person classes	263	14.8
	Online classes	116	6.5
	No classes	294	16.6
Third	In-person classes	370	20.9
	Online classes	311	17.5
	No classes	420	23.7
Primiparous			
Yes	In-person classes	370	20.9
	Online classes	298	16.8
	No classes	190	10.7
No	In-person classes	263	14.8
	Online classes	129	7.3
	No classes	524	29.5

**Table 2:** The association between studied factors and the reported levels of STAI-State and EPDS. Statistically significant results are in bold.

	Anxiety (STAI-State)			Depression (EPDS)		
Factors	Mean ± SD	ηp^2^	*p*	Mean ± SD	ηp^2^	*p*
Pregnancy complications		0.004	0.011		0.004	0.011
Yes	43.84 ± 10.80			7.02 ± 3.94		
No	46.28 ± 10.71			8.06 ± 5.15		
COVID-19 infection		0.001	0.291		0.001	0.341
Yes	43.44 ± 9.25			7.78 ± 4.86		
No	44.44 ± 10.99			8.27 ± 5.17)		
Trimester of pregnancy		0	0.413		0.003	0.032
Third	43.59 ± 10.71			7.98 ± 5.08		
Second	44.93 ± 11.01			8.43 ± 5.21		
Parity		0.002	0.07		0.001	0.34
Primiparas	43.34 ± 10.81			7.95 ± 5.08		
Multiparas	45.36 ± 10.92			8.27 ± 5.21		
	**β**			**β**		
Maternal age	−0.59	0.002	0.067	0.74	0.003	0.041
STAI-Trait	1.43	0.48	<0.001			

## Data Availability

The data presented in this study are available on request from the corresponding author. The data are not publicly available due to privacy restrictions.
